# Temporal development and neutralising potential of antibodies against SARS-CoV-2 in hospitalised COVID-19 patients: An observational cohort study

**DOI:** 10.1371/journal.pone.0245382

**Published:** 2021-01-26

**Authors:** Isa Murrell, Donall Forde, Wioleta Zelek, Linda Tyson, Lisa Chichester, Nicki Palmer, Rachel Jones, B. Paul Morgan, Catherine Moore

**Affiliations:** 1 Wales Specialist Virology Centre, Public Health Wales Microbiology, University Hospital of Wales, Cardiff, Wales, United Kingdom; 2 Systems Immunity Research Institute, School of Medicine, Cardiff University, Cardiff, Wales, United Kingdom; Ramathibodi Hospital, Mahidol University, THAILAND

## Abstract

Antibody responses are important in the control of viral respiratory infection in the human host. What is not clear for SARS-CoV-2 is how rapidly this response occurs, or when antibodies with protective capability evolve. Hence, defining the events of SARS-CoV-2 seroconversion and the time frame for the development of antibodies with protective potential may help to explain the different clinical presentations of COVID-19. Furthermore, accurate descriptions of seroconversion are needed to inform the best use of serological assays for diagnostic testing and serosurveillance studies. Here, we describe the humoral responses in a cohort of hospitalised COVID-19 patients (n = 19) shortly following the onset of symptoms. Commercial and ‘in-house’ serological assays were used to measure IgG antibodies against different SARS-CoV-2 structural antigens–Spike (S) S1 sub-unit and Nucleocapsid protein (NP)–and to assess the potential for virus neutralisation mediated specifically by inhibition of binding between the viral attachment protein (S protein) and cognate receptor (ACE-2). Antibody response kinetics varied amongst the cohort, with patients seroconverting within 1 week, between 1–2 weeks, or after 2 weeks, following symptom onset. Anti-NP IgG responses were generally detected earlier, but reached maximum levels slower, than anti-S1 IgG responses. The earliest IgG antibodies produced by all patients included those that recognised the S protein receptor-binding domain (RBD) and were capable of inhibiting binding to ACE-2. These data revealed events and patterns of SARS-CoV-2 seroconversion that may be important predictors of the outcome of infection and guide the delivery of clinical services in the COVID-19 response.

## Introduction

Severe Acute Respiratory Syndrome Coronavirus 2 (SARS-CoV-2) emerged in Wuhan, China, in late 2019, followed by rapid global spread with coronavirus-disease-2019 (COVID-19) declared a pandemic on 11^th^ March 2020 [1, 2]. As of 30^th^ October 2020, 44,592,789 laboratory-confirmed cases and 1,175,533 deaths globally have been reported to WHO [3]. Due to the recent emergence of this virus, there exist substantial gaps in knowledge of the interactions between SARS-CoV-2 and the human host. In particular, greater understanding of immune responses to SARS-CoV-2 is required to reveal predictors to the clinical outcome of infection, and to inform strategies for the management of patients and prevention of onward transmission. Importantly, correlates of immune control will also guide the development and assessment of candidate vaccines.

SARS-CoV-2 belongs to the *Coronaviridae* family of single-stranded, positive-sensed RNA viruses, and is the seventh member that causes infection in humans [2, 4]. Four human coronaviruses (hCoVs)—hCoV-229E, hCoV-OC3, hCoV-NL63, and hCoV-HKU1—circulate globally and are associated with seasonal mild respiratory infection [5–7]. Two coronaviruses are associated with more severe infection; MERS-CoV that remains largely a zoonotic infection associated primarily with exposure to camels, and SARS-CoV that emerged in 2002, though was eliminated in 2003 following concerted global efforts [8–10]. The clinical presentations of SARS-CoV-2 range in severity, from mild or even asymptomatic, to severe disease that can include pneumonia, bronchitis, and acute respiratory distress syndrome (ARDS) [11]. Compared to SARS-CoV and MERS-CoV, SARS-CoV-2 has a lower case-fatality rate, yet more efficient person-to-person transmission.

Immune responses to coronaviruses include the generation of antibodies against viral structural proteins, with nucleocapsid protein (NP) and spike glycoprotein (S) being the dominant antigens and principal targets for SARS-CoV-2 serological assays [12, 13]. Importantly, the S glycoprotein has a dual role as a viral attachment protein (VAP) and fusion protein, facilitating virus binding and entry via the angiotensin-converting enzyme 2 (ACE-2) found on epithelial cells lining the upper respiratory tract, lungs and gastric system [14–19]. Interactions with ACE-2 are mediated by the receptor-binding domain (RBD) located in the S1 sub-unit of the S protein, and antibodies specific for epitopes within the RBD contribute to the neutralisation of SARS-CoV-2 [20–23]. The robust neutralising effect of these antibodies impedes virus replication and plays a vital role in reducing viral loads and subsequent clearance of infection [24].

The timescale between the onset of symptoms and production of anti-SARS-CoV-2 antibodies (seroconversion) has been described to vary from person-to-person, likely influenced by original inoculation dose and disease severity [24–27]. Prior to the production of protective antibodies, however, virus replication may occur unchallenged, with onward transmission more likely to occur [28, 29]. Hence, clearly defining the timescale for the development of antibodies with protective potential will be critical to inform strategies for successful intervention.

We describe the humoral immune responses to SARS-CoV-2 seroconversion amongst a cohort of hospitalised COVID-19 patients from whom serial sera had been collected early following diagnosis. This was achieved using a combination of commercial and ‘in-house’ serological assays targeting NP and S protein S1 sub-unit, and specifically the RBD of S protein. To assess the RBD:ACE-2 binding inhibition potency of the detected antibodies we used a novel commercial assay utilising a soluble RBD that binds to ACE-2 coated on plates [30]. Data from this work was used to inform the best use of serological testing in the management of patients, and to determine the utility of serology in the broader clinical and health protection services for the COVID-19 response in Wales.

## Materials and methods

### Ethics statement

The work described was undertaken as part of an evaluation/verification of serological assays that detect anti-SARS-CoV-2 antibodies for routine service delivery, and as such, was defined by the Cardiff and Vale (CAV) Health board ethics committee as ‘service development’. Ethical approval and patient consent were not required. The authors did not participate in the collection of the serum samples. After gathering basic information, including gender, age, known comorbidities, reported date of onset and outcome, samples were anonymised as part of a single panel to test across multiple assays. All testing was conducted retrospectively, and the results from this study were not used to guide the management of the study cohort patients.

### COVID-19+ patient serum samples

Residual serum samples collected from laboratory confirmed (RT-PCR) COVID-19 inpatients (March to May 2020) were obtained from the Blood Sciences department at the University Hospital of Wales (UHW), Cardiff, prior to discard.

### RBD ELISA and sVNT assay sensitivity and specificity panels

The sensitivity and specificity panels used during optimisation of the SARS-CoV-2 S protein receptor-binding domain (RBD) ELISA included residual sera previously shown to be positive (n = 29) or negative (n = 30) for anti-S1 IgG by the EuroImmun assay.

The specificity panel used for the surrogate virus neutralisation test (sVNT) included residual serum samples previously shown to be anti-S1 IgG negative (n = 19). To demonstrate the lack of cross-reactivity with antibodies specific to other coronaviruses, this panel included sera from patients with recent seasonal-hCoV infection (n = 10); these were collected at least 14 days following RT-PCR confirmation of seasonal hCoV infection. All four seasonal coronaviruses were represented (NL63 n = 3, HKU1 n = 2, OC43 n = 4, 229E n = 1). In all cases, SARS-CoV-2 was ruled out by RT-PCR.

### Serological testing for IgG specific for the major SARS-CoV-2 antigens

Detection of IgG antibodies against the major SARS-CoV-2 structural protein antigens was performed using CE-marked assays verified as suitable for diagnostic testing as part of the routine serological services offered at Wales Specialist Virology Centre (WSVC) [31].

#### SARS-CoV-2 nucleocapsid protein (NP) CMIA

Antibodies against the SARS-CoV-2 nucleocapsid protein (NP) were detected by the Abbott IgG chemiluminescent micro-particle immunoassay (CMIA) on the fully automated, random-access, Abbott Architect platform (Abbott, Maidenhead, UK). This assay displayed 90.24% sensitivity (95% CI: 75.20–97.06) and 100% specificity (95% CI: 95.20–100) during verification at WSVC.

#### SARS-CoV-2 spike (S) protein S1 sub-unit ELISA

Antibodies against the SARS-CoV-2 spike (S) protein S1 sub-unit were detected by the EuroImmun IgG ELISA (EuroImmun AG, Lubeck, Germany) using the DS2 plate auto-analyser (Dynex Technologies Ltd, Worthing, UK). This assay displayed 87.8% sensitivity (95% CI: 73.80–95.92) and 100% specificity (95% CI: 88.43–100) during verification at WSVC.

### SARS-CoV-2 S protein receptor-binding domain (RBD) ELISA

Antibodies against the SARS-CoV-2 S protein RBD were detected by an ‘in-house’ direct ELISA based on a published method [32, 33]. Maxisorp (Nunc, Loughborough, UK) 96-well plates were coated with RBD protein (2 μg/mL) in bicarbonate buffer (pH 9.6) at 4°C overnight. On the day of assay, wells were blocked with 3% (w/v) non-fat dried milk powder (Sigma Aldrich, # 70166-500G) in phosphate-buffered saline containing 0.1% Tween 20 (PBS-T) for 1 hour (hr) at room temperature (RT). Dilutions of patient sera (1 in 50 in 1% Milk PBS-T) were added to wells and incubated for antibody:RBD binding to occur (2 hr, RT). After washing with PBS-T, bound antibody was detected with secondary antibody (donkey anti-human IgG F(ab’)_2_-horseradish peroxidase (HRP); #709-036-149, Jackson ImmunoResearch, Ely, UK; 1 hr RT), with reactions developed using O-phenylenediamine dihydrochloride (OPD, SIGMAFASTTM; Sigma-Aldrich, # P9187-50SET). Absorbance (OD 492 nm) was measured in each well. Then mean background signal from two ‘blank’ wells was subtracted from the sample signals reported. A cut-off of 0.7 OD was applied to distinguish negative/positive seroreactivity reported by the RBD ELISA.

### SARS-CoV-2 RBD:ACE-2 binding inhibition

The RBD:ACE-2 binding inhibition potency of serum samples was investigated by a plate-based SARS-CoV-2 surrogate virus neutralising test (sVNT) (GenScript, New Jersey, USA). In brief, serum samples were mixed with soluble SARS-CoV-2 RBD-horse-radish peroxidase conjugates (sRBD-HRP) and incubated for antibody:sRBD-HRP binding to occur. Mixtures were then added to wells coated with ACE-2, and plates were further incubated for sRBD-HRP:ACE-2 binding to occur. Unbound sRBD-HRP was washed from the wells, and reactions were developed with 3,3’,5,5’-tetramtheylnezdinehttps://en.wikipedia.org/wiki/3,3%27,5,5%27-Tetramethylbenzidine (TMB). Absorbance (OD 450 nm) was measured in each well. The percent (%) sRBD-HRP:ACE-2 binding inhibition was calculated as:

$$\left(1-(\frac{Sample\;OD}{negative\;control\;OD})\right)X\;100$$

An ‘in-house’ determined cut-off of 25% RBD:ACE-2 binding inhibition was applied to sVNT assays.

### Statistical analysis

Statistical analyses were performed in Prism v7 (Graphpad). Best-fit curve interpolation analysis was performed by sigmoidal 4PL regression, with 95% CI determined from the mean signals. Seroconversion detection rates were analysed by Kaplan-Meier plots. For correlation analyses, data were first checked for Gaussian distribution by D’Agostino and Pearson’s normality test, before performance of either Pearson’s parametric or Spearmans’s non-parametric correlation test. The significance of any observed correlation was reported by two-tailed p test.

## Results

### Study cohort

Patients were recruited to the study cohort in a random, non-biased approach, based solely on the availability of sequential sera spanning days 7–14 after laboratory confirmation (by RT-PCR) of SARS-CoV-2. Initially, 21 patients were identified. Symptom onset dates (set as day 0) were used to construct infection course timelines for each patient. However, these details were not available for two patients who were subsequently excluded.

Demographic characteristics of the patient cohort are described in Table 1. Eleven (58%) of the 19 of the patients were female, eight (42%) were male, and mean age was 70 years. The majority of patients (84%) had clinically relevant co-morbidities (described in S1 Table). The mean duration of inpatient stay was 29·5 days, with seven (37%) patients requiring admission to the critical care unit (CCU); four of which were male (50% of all male patients) compared to three female (~27% of all female patients). Three patients died (two males, one female) giving an overall mortality rate of 16% amongst the patient cohort.

**Table 1:** Baseline characteristics of the cohort patients.

	Gender (M/F^a^)	Age (years)	Co-morbid (Y/N ^b^)	Inpatient duration (days)	CCU^c^ admission (Y/N ^b^)	Mortality (Y/N ^b^)
*Patient 1*	M	78	Y	50	N	N
*Patient 2*	M	72	Y	17	N	N
*Patient 3*	M	68	Y	20	Y	Y
*Patient 4*	F	65	Y	30	Y	N
*Patient 5*	M	65	Y	41	N	N
*Patient 6*	F	62	Y	19	Y	N
*Patient 7*	F	86	Y	65	N	N
*Patient 8*	M	59	Y	26	Y	Y
*Patient 9*	M	32	N	25	Y	N
*Patient 10*	F	52	Y	35	Y	Y
*Patient 11*	M	34	Y	17	Y	N
*Patient 12*	F	78	Y	36	N	N
*Patient 13*	F	86	N	27	N	N
*Patient 14*	F	91	Y	25	N	N
*Patient 15*	F	88	Y	57	N	N
*Patient 16*	F	75	Y	20	N	N
*Patient 17*	F	93	Y	16	N	N
*Patient 18*	M	78	Y	24	N	N
*Patient 19*	F	72	Y	11	N	N
	***Summary***					
	**n(%)**^d^	**mean(med)**^e^	**n(%)**^d^	**mean(med**^e^**)**	**n(%)**^d^	**n(%)**^d^
	8(42)/11(58)	70(72)	3(16)/16(84)	29.5 (25)	7(37)/12(63)	3(16)/16(84)

^a^M = male, F = female

^b^Y = yes, N = no

^c^CCU = critical care unit

^d^n(%) = number(percentage)

^e^med = median

### Temporal development of anti-S protein S1 sub-unit and anti-nucleocapsid protein (NP) IgG antibodies following the onset of COVID-19 symptoms

Both commercial assays used to explore anti-S1 IgG and anti-NP IgG are described as semi-quantitative. Thus, whilst no titres are reported, antibody levels could be inferred by the degree of reactivity observed (reported as ratios of sample/calibrator read-outs). This was exploited to determine the temporal development of antibody responses against the respective antigens.

**Fig 1:**
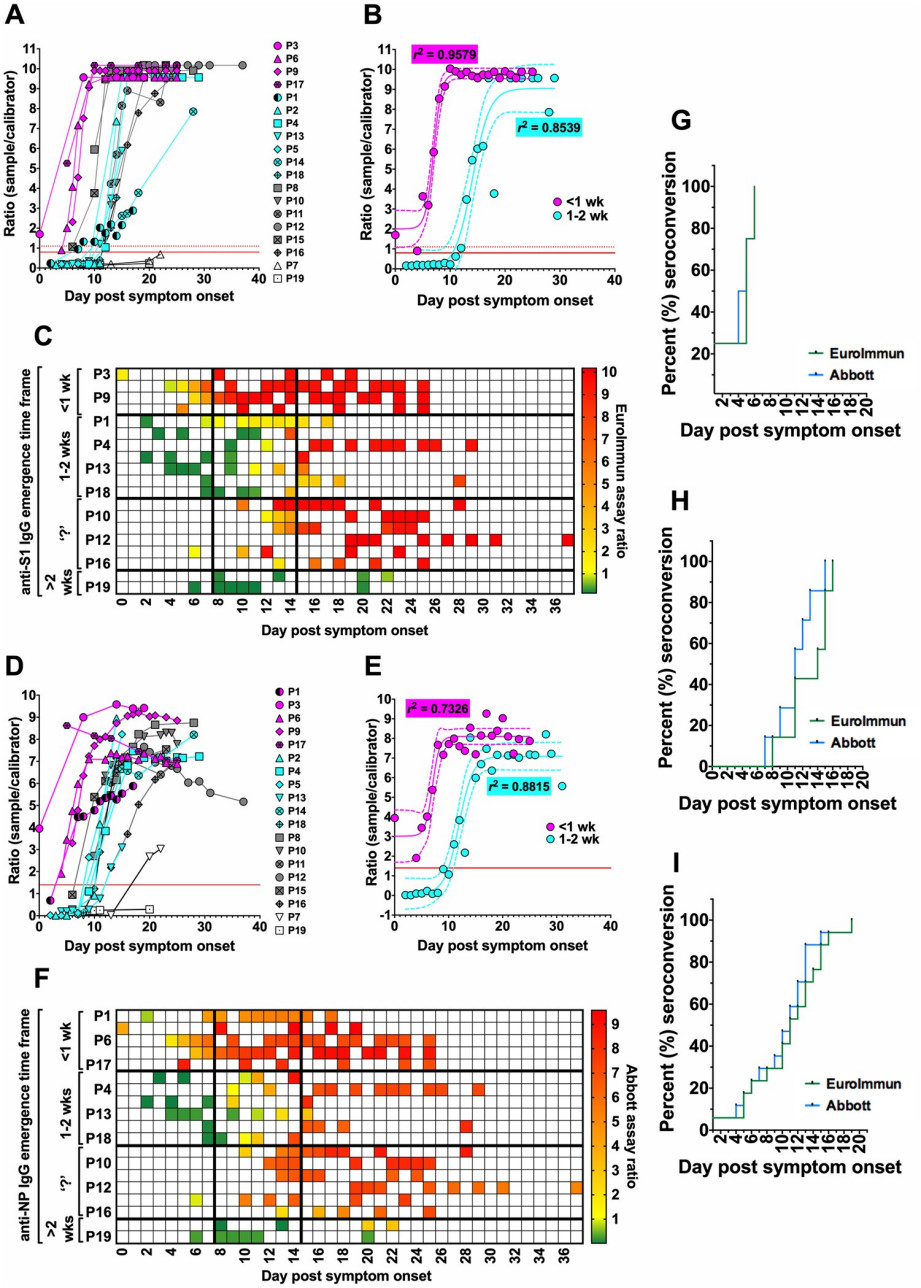
Temporal emergence of anti-S1 and anti-NP IgG amongst the cohort members. Serum samples collected from the cohort members over time were analysed for IgG against the major SARS-CoV-2 antigens. (**A**) Individual time course data for anti-S1 IgG emergence, and **(B)** best-fit curves for anti-S1 IgG responses occurring after < 7 days or between 7–14 days pso. Cut-offs for negative (ratio 0.8) and positive (ratio 1.1) seroreactivity in the EuroImmun assay are depicted by solid red line and dashed red line, respectively. (**C**) Data from **A** presented as a heat-map. (**D**—**F**) As in **A-C**, but for anti-NP IgG emergence. The cut-off (ratio 1.4) for negative/positive seroreactivity in the Abbott assay depicted in solid red line. Patient #1 was considered an outlier and was therefore excluded from all best-fit curve analyses. In **B** and **E**—dashed trend lines indicate 95% CI ranges; and *r*^2^ values describe tightness of fit. In **C** and **E**—‘?’ indicates undetermined seroconversion time frame. Kaplan-Meier curves comparing the seroconversion detection by the EuroImmun and Abbott assays amongst (**G**) the seroconverted, (**H**) those where seroconversion was observed, and (**I**) all cohort members combined.

Anti-S1 IgG seroconversion was observed for 17/19 patients, though occurring with noticeably variable kinetics (Fig 1A). For four patients, measurable anti-S1 IgG developed within 7 days post symptom onset (pso), whilst for seven patients it clearly occurred between 7–14 days. Strikingly, the anti-S1 IgG response in patient #1 that became measurable between days 7–14 pso did not progress in the same way as seen for all other patients. For patient #15, measurable anti-S1 IgG developed between days 6–10 pso. It was impossible to determine when this seroconversion occurred in five of the patients, due to sera being collected later relative to symptom onset dates and seroreactivity in the very first samples. No anti-S1 IgG response was observed for patients #7 or #19. Best-fit curves (Fig 1B) and heat-maps (Fig 1C) illustrate the different anti-S1 IgG evolution kinetics amongst the cohort.

Similar patterns were observed for anti-NP IgG seroconversion (Fig 1D). Patients with rapid anti-S1 IgG responses each developed measurable anti-NP IgG within 7 days pso. Patient #1 also seroconverted to anti-NP IgG within 7 days (earlier than anti-S1 IgG), but this response too demonstrated stunted progression. The majority of the remaining patients with later anti-S IgG responses also produced anti-NP IgG slightly earlier, yet still first detectable after 7 days. In contrast, anti-NP IgG was detected later compared to anti-S IgG for patient #13. Interestingly, in seven patients, including all four rapid seroconverts, anti-NP IgG levels decreased as infection time courses progressed. Of note, whilst no anti-S1 IgG response was seen for patient #7, anti-NP IgG was detected late into the infection time course. No anti-NP IgG seroconversion was observed for patient #19. Best-fit curves (Fig 1E) and heat-maps (Fig 1F) illustrate the different anti-NP IgG kinetics.

In patients with earlier antibody responses, maximum anti-S1 IgG assay signals were achieved on average by day 10 pso (Fig 1B), whilst maximum anti-NP IgG signals were achieved by day 16 (Fig E). In patients with delayed responses, anti-S1 IgG became detectable on average around day 11 pso with maximum signals after day 21 (Fig 1B), whilst anti-NP IgG became detectable around day 10 with maximal signals after day 20 (Fig 1E). Overall, seroconversion occurred more rapidly to NP than S1, and this was most apparent in the patients with delayed responses (Fig 1G–1I).

### Evolution of anti-RBD IgG following the onset of COVID-19 symptoms

**Fig 2:**
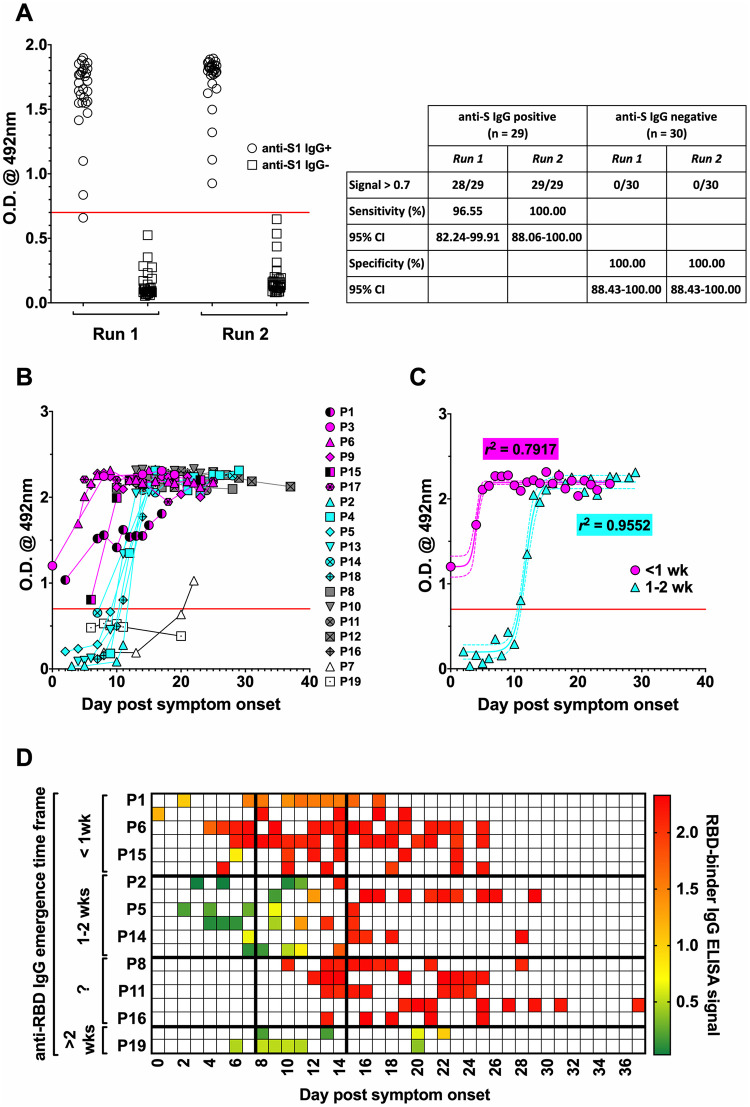
Temporal emergence of anti-RBD IgG antibodies. Serum samples collected from the cohort members over time were analysed for anti-RBD IgG by an ‘in-house’ ELISA. (**A**) The anti-RBD IgG ELISA was optimised by assaying sera previously shown to be either positive (n = 29) or negative (n = 30) for anti-S1 IgG by the EuroImmun assay; each serum was assayed in duplicate (means are depicted), and the optimisation was based on data from two separate runs. The calculated sensitivity and specificity of the anti-RBD IgG ELISA are described in the table. (**B**) Individual time-course data and (**C**) best-fit curves for the emergence of RBD-binding IgG occurring after < 7 days or between 7–14 days pso. In solid red line is the cut-off (0.7) for negative/positive seroreactivity. Patients #1 and #15 were considered outliers and were therefore excluded from best-fit curve analyses. In **C**–dashed lines indicate 95% CI ranges; *r*^2^ values describe tightness of fit. (**D**) Data from **B** presented as a heat-map. ‘?’ indicates undetermined seroconversion time frame.

Preliminary optimisation work to determine a suitable cut-off for the RBD ELISA assay involved exploring the signals reported for sera shown to be negative (n = 30) or positive (n = 29) for anti-S1 IgG; this was performed over two independent runs. An appropriate cut-off was initially determined as the mean signal from the S1 IgG negative samples + 3SD, calculated as 0.5–0.6 OD units over the repeat runs. This cut-off was further adjusted to 0.7 OD units in favour of assay specificity (100%) over sensitivity (96.55–100%) (Fig 2A).

Under these optimised assay parameters, the anti-RBD IgG responses amongst the cohort closely matched the previously seen anti-S1 IgG responses (Fig 2B). The patients who developed measurable anti-S1 IgG within 7 days pso demonstrated equally rapid anti-RBD IgG kinetics, whilst for the majority of patients with anti-S1 IgG first measurable after day 7, this was also mirrored by equally delayed anti-RBD IgG kinetics. Noteworthy, anti-RBD IgG was detectable earlier (<7 days pso) in patients #1 and #15 compared to anti-S1 IgG (> 7 days). Furthermore, the anti-RBD-IgG response in patient #1 evolved slowly, similarly to anti-S1 IgG and anti-NP IgG. Patient #18 also had an earlier anti-RBD IgG response compared to anti-S1 IgG, though still detectable only after 7 days. Only patient #19 developed no measurable anti-RBD IgG, concordant with their anti-S1 IgG response.

Best-fit curve analysis (Fig 2C) and heat-maps (Fig 2D) revealed a good concordance between anti-RBD development and the previously observed S1-IgG kinetics.

### RBD:ACE-2 binding inhibition capacity of antibodies produced during SARS-CoV-2 seroconversion

**Fig 3:**
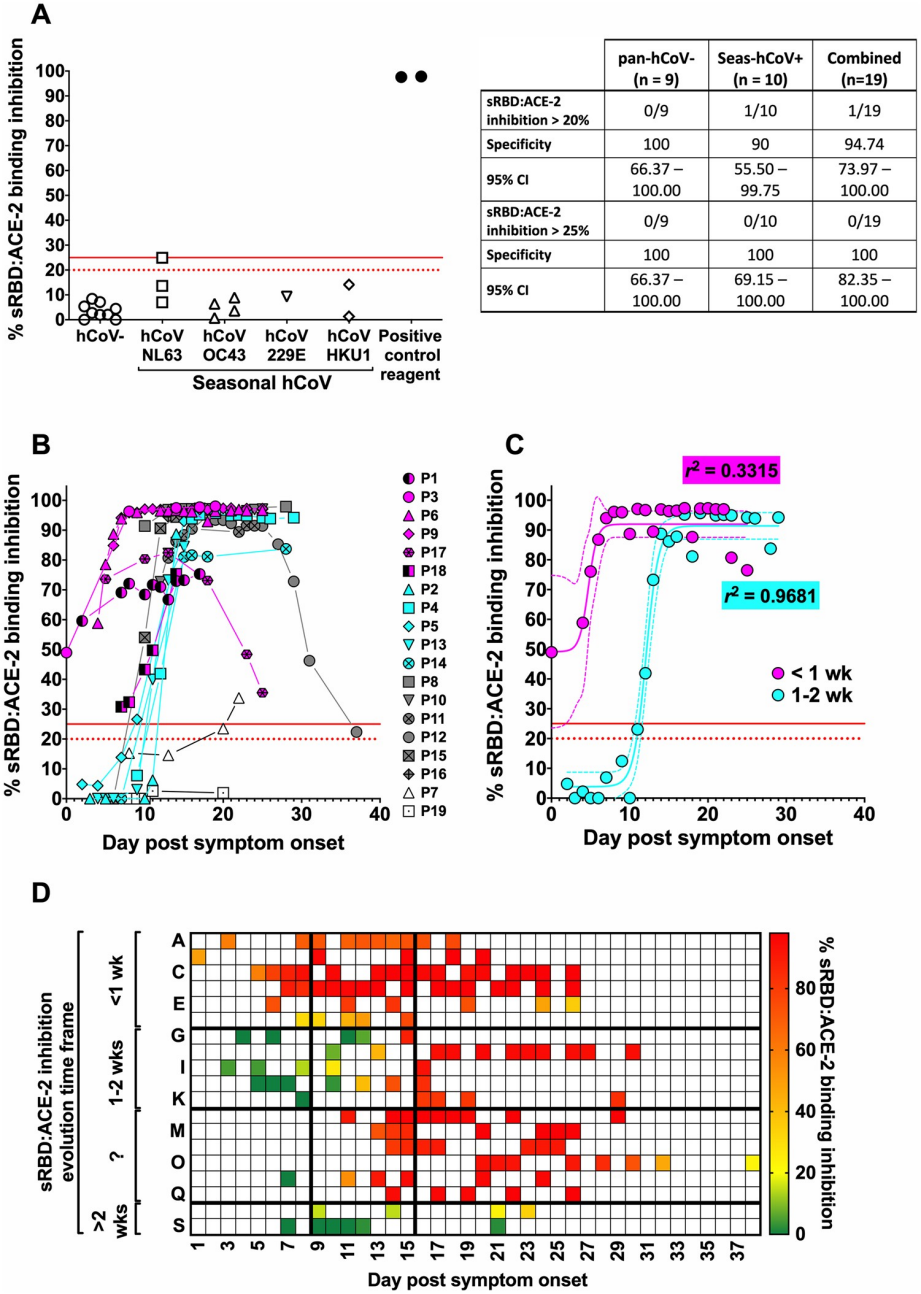
RBD:ACE-2 binding inhibition potency of antibodies produced over seroconversion. Serum samples collected from the cohort members following the onset of symptoms were analysed for RBD:ACE-2 binding inhibition potency by the GenScript surrogate Virus Neutralisation Test (sVNT). (**A**) An assessment of the assay specificity was conducted using hCoV seronegative sera (n = 9) and seasonal hCoV sera (n = 10). The calculated sensitivity and specificity of the anti-RBD IgG ELISA are described in the table. (**B**) Individual time-course data and (**C**) best-fit curves for RBD:ACE-2 inhibition evolving after < 7 days or between 7–14 days pso. In **B+C**–the manufacturer’s suggested sVNT assay cut-off (20% sRBD:ACE-2 binding inhibition relative to the negative control) is indicated by broken red line, and the ‘in-house’ applied cut-off (25%) is indicated by solid red line. Patients #1 and #18 were considered outliers and were therefore excluded from best-fit curve analyses. In **C**–dashed lines indicate 95% CI ranges; *r*^2^ values describe tightness of fit. (**D**) Data from **B** presented as a heat-map. ‘?’ indicates undetermined seroconversion time frame.

Verification of the commercial surrogate virus neutralisation test (sVNT) specificity was based on an assessment of the background RBD:ACE-2 inhibition potency of samples previously shown to be negative for anti-S1 IgG (n = 19). Under the manufacturer’s suggested assay cut-off of 20% RBD:ACE-2 binding inhibition, no positive results were produced for any pan-hCoV seronegative sample (n = 9) produced, nor nine sera from seasonal hCoV positive patients (n = 10) (Fig 3A). However, low-level positivity was seen for one seasonal hCoV serum sample, and specificity of the sVNT was therefore estimated at 94.74%. An ‘in-house’ applied cut-off was determined from the mean control signal +3SD and calculated at 24.83%, and an adjusted cut-off of 25% was applied during testing of the cohort samples in favour of greater assay specificity (100%). Positive controls supplied with the kit demonstrated >97% sRBD:ACE-2 binding inhibition over all runs.

Results from sVNT neutralisation assays broadly reflected the anti-RBD IgG responses (Fig 3B). In most patients who developed detectable anti-RBD IgG within 7 days pso, this was matched by rapid evolution of antibodies with >90% RBD:ACE-2 binding inhibition potency within similar time. However, this same robust protective response was not seen for patient #17 for whom RBD:ACE-2 inhibition potency peaked at ~80%, before a sharp decline throughout the remaining sample series. The stunted anti-RBD IgG development seen for patient #1 was matched by slowly evolving RBD:ACE-2 binding inhibition potency that did not exceed ~85%. For patient #15 who had also demonstrated rapid anti-RBD IgG kinetics, there was a delay in in the detection of RBD:ACE-2 inhibition. The patients with anti-RBD IgG response first detected after >7 days pso also displayed delayed RBD:ACE-2 binding inhibition evolution. Interestingly, this inhibition was detected in patients #5, #7 and #18 earlier than anti-S1 IgG or anti-RBD IgG. Also noteworthy, the samples collected from patient #12 commenced with high blocking ability (>90%), yet this reduced rapidly in sera collected from day 25 onwards.

On average, sRBD:ACE-2 inhibition potency in those with rapid immune responses reached maximum measurable levels between day 9–10 pso, whilst for those with delayed responses, RBD:ACE-2 inhibition evolved from around day 10 and reached maximal levels at around day 19 (Fig 3C and 3D).

### Correlation between the humoral response and RBD:ACE-2 binding blocking capacity

**Fig 4:**
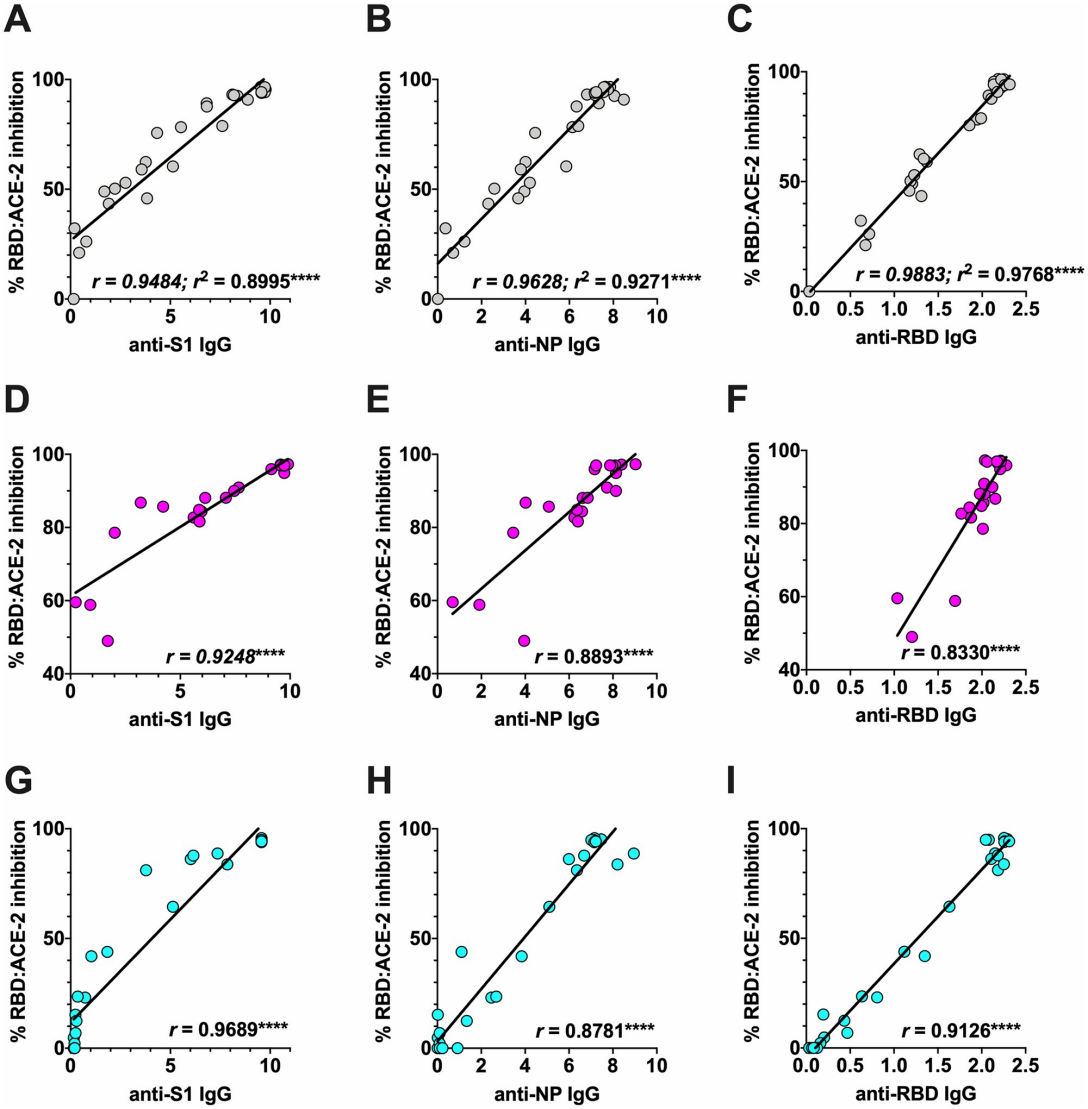
Correlates of RBD:ACE-2 binding inhibition potency. The relationship between anti-S1 IgG, anti-NP IgG or anti-RBD IgG levels and sRBD:ACE-2 inhibition potency was investigated by Pearson’s correlation test (when data displayed Gaussian distribution) or by Spearman’s nonparametric correlation test (when data were not normally distributed), with the significance of any correlation reported by two-tailed p test. (A-C) Correlation between anti-S1 IgG, anti-NP IgG and anti-RBD IgG to RBD:ACE-2 binding inhibition potency across the cohort (excluding patients 12 and 17). (D-E and F-H) As in **A-C**, though describing correlates specifically for cohort members that seroconverted with 7 days pso, or after 7 days pso, respectively. Abbreviations/symbols: *r* describes correlate coefficients; *r*^2^ describes the coefficient of determination (for Pearson’s correlation test only); **** p ≤ 0.0001.

The different seroconversion markers investigated in this study could each be significantly correlated with RBD:ACE-2 inhibition potency in the majority of individual patients (S1, S2 and S3 Figs). Following exclusion of patients #12 and #17 that each displayed dramatic loss of RBD:ACE-2 binding inhibition potency as their respective infection time-courses progressed, the strongest association across the remaining cohort members was seen for anti-RBD IgG, followed by anti-NP IgG and then anti-S1 IgG (Fig 4A–4C).

To explore any difference in these correlations according to response kinetics, the cohort patients were assigned to groups based on conclusive evidence of early seroconversion (positivity for each of anti-S1 IgG, anti-NP IgG, anti-RBD IgG and RBD:ACE-2 inhibition prior to day 7) (Fig 4D–4F), or delayed seroconversion (positivity in the same assays clearly developing after 7 days) (Fig 4G–4I). In both the ‘early’ and ‘delayed’ humoral response groups, anti-S1 IgG was best correlated with RBD:ACE-2 inhibition. However, in the ‘early’ responder group, anti-NP IgG levels were correlated with RBD:ACE-2 binding inhibition potency more significantly than anti-RBD IgG levels, yet in the delayed responder group, anti-RBD IgG levels were correlated more significantly than anti-NP IgG levels.

## Discussion

COVID-19 reached pandemic level soon after the emergence of SARS-CoV-2, and currently remains the priority healthcare challenge globally. Although incidence has reduced since the implementation of social restrictions, future waves of infection are anticipated as these measures are inevitably eased [34]. Hence, there is an urgent need to understand immune responses to SARS-CoV-2. The findings we describe contribute to the growing data regarding humoral responses to SARS-CoV-2 and provide novel insights into the protective potential of antibodies produced during seroconversion.

The cohort patients in our study appeared to fall within distinct temporal humoral responder categories, with very early responses seen in some, through to very late responses seen in others. However, major limitations in this categorisation are that the different seroconversion time frames could simply reflect inaccuracies in reported dates of symptom onset, or differences in incubation period for each infection (estimated on average 5 days, but anywhere between 2–14 days) [35]. Nonetheless, similar variation in COVID-19 humoral response kinetics have been described elsewhere [25–27, 36]. There exist few reports describing humoral-response kinetics during seroconversion to seasonal coronaviruses. One study, based on the challenge of individuals with hCoV-229E, described antibody emergence on average around day 8 post exposure and peaking at day 14 [37]. For SARS-CoV and MERS-CoV infection, IgG seroconversion is reported anywhere between one to several weeks after symptom onset [38–42]; similar to what was observed in our cohort.

One potential explanation for the apparent ‘early’ and ‘late’ responses in this study may be that the assays used target IgG, whilst Long and co-workers previously described alternative SARS-CoV-2 seroconversion dominated by IgM [26]. It is therefore feasible that ‘early’ and ‘late’ immune responses described here reflect IgG and IgM dominant seroconversion, respectively. The assays that we utilised appeared mostly un-affected by cross-reactive antibodies from seasonal hCoV infection, albeit based on a relatively small panel. Many published investigations have reported only little cross-reactivity for antibodies against NP, and negligible-to-zero cross-reactivity for the spike protein S1 sub-unit and RBD [43, 44]. However, one study does report cross-reactivity of antibodies recognising assembled S protein trimers, though the presence of the S protein S2 sub-unit that is more conserved amongst different coronaviruses compared to the S1 sub-unit is thought to be responsible [45]. In light of this, it seems most plausible that any cross-reactive antibodies may have impacted anti-NP IgG assays, but not anti-S1 IgG, anti-RBD IgG, or RBD:ACE-2 inhibition assays. The low-level RBD:ACE-2 inhibition displayed by hCoV-NL63+ serum in the sVNT is curious in this regard. However, this serum was from a patient with acute lymphoblastic leukaemia (ALL), and it is possible that non-specific reactivity detected was due to deranged haematological features of the donor.

Earlier detection of anti-NP IgG implies this may be the most suitable seroconversion marker for diagnostic and serosurveillance purposes, and mirrors observations reported for SARS-CoV-2, and also SARS-CoV [36, 46]. Aside from different analytical sensitivities of the assays used [28], one possibility for later anti-S1 IgG detection is that the extensive glycosylation of S1 antigen may impede antibody responses. This may also explain the more sensitive detection of anti-RBD IgG compared to anti-S1 IgG, since the RBD is less densely glycosylated [47]. Furthermore, S1 sub-unit exists in ‘up/open’ and ‘down/closed’ conformations in the S protein trimer incorporated into virions [48–50], and antibodies recognising epitopes in alternative confirmations are described [51]. Thus, the conformation of S1 sub-unit used in the EuroImmun assay may also restrict accessibility to epitopes targeted during natural infection. Still, the concurrent detection of anti-RBD IgG that may be protective role is a clear benefit of anti-S1 IgG assay. Furthermore, anti-S1 IgG levels appeared more stable than anti-NP IgG, and thus may have a broader window for detection.

The different seroconversion markers assayed could be correlated with RBD:ACE-2 inhibition potency, implying that each has potential utility as prognostic markers as our understanding of SARS-CoV-2 immune control increases. Most curious is the correlation with anti-NP IgG levels. The potent humoral antigenicity of SARS-CoV NP has previously been hypothesised, with NP peptides predicted to be available for B cell activation following release from infected cells during cellular immune responses [52]. However, the functional role of anti-NP IgG is unclear, with any contribution to neutralisation unlikely, given that NP is contained internally within virions. Instead, roles for anti-NP IgG by Fc-mediated mechanisms have been speculated. Hence, correlations between anti-NP IgG and RBD:ACE-2 binding inhibition likely reflects an indirect relationship between different humoral response elements developing in parallel. With this in mind, it is intriguing that the two patients in whom RBD:ACE-2 binding inhibition potency decreased also had concomitant decreases in anti-NP IgG levels. Correlations between RBD:ACE-2 inhibition and anti-S1 IgG or anti-RBD IgG levels are perhaps easier to consider, since anti-S1 IgG encompass anti-RBD IgG, a subset of which inhibit binding to ACE-2. Investigations exploring concordance between the EuroImmun IgG assay, anti-RBD IgG ELISA, and the sVNT assay have previously been reported [53, 54]. Bond et al reported broadly equivalent sensitivity between the EuroImmun assay (93.8%) and sVNT (91.4%) using sera collected >14 days pso, whilst McGregor et al reported perfect concordance with anti-RBD ELISA and sVNT positivity using sera collected > 7 days pso. Direct comparison to our findings is difficult, since the exact time points pso at which samples used in these works were acquired are not available. Nevertheless, high concordance between these assays is reported here, and any discordance observed in our studies was confined to the primary samples in any given series.

Our descriptions of SARS-CoV-2 seroconversion build on those of To and co-workers [25], but reveal new insights into the temporal development of antibodies that could contribute to neutralisation. The demonstration that anti-RBD IgG with neutralising potential develop early after the onset of symptoms is encouraging, and suggests immune control may commence soon following infection. Indeed, the early emergence of neutralising potency following symptom onset has been described elsewhere [55]. However, whilst our investigations of neutralising potential focussed specifically on antibodies that inhibit RBD and ACE-2 binding, not all neutralising antibodies will be revealed by this approach. A recently described mechanism of SARS-CoV-2 neutralisation involves antibodies cross-linking epitopes of the S1 sub-unit ‘down/closed’ conformation, thereby locking the S-protein trimer in a prefusion state, and ultimately preventing RBD:ACE-2 interactions [51]. The RBD fragments used in the sVNT may be refractory to ‘prefusion locking’, hence there is obvious scope to underreport the neutralisation potential of sera by the sVNT. Clearly, correlates of neutralisation in the context of live virus will undoubtedly be more meaningful.

The focus on anti-SARS-CoV-2 neutralising antibodies (nAbs) is borne from understanding of the mechanisms of immune control against other coronaviruses [56]. Two protective activities of nAbs are suggested: i) the prevention of cell-entry to impede intra-host spread and pathogenesis; and ii) interference in the cell-entry of shed virus to reduce inter-host transmission. Considering the protective role of nAbs, the decline in neutralising potency observed for two of the cohort members raises concerns regarding the longevity of antibody-mediated immunity to SARS-CoV-2. There are seemingly conflicting reports regarding the longevity of anti-SARS-CoV-2 antibodies, with waning of IgG levels during the early convalescent phase described for both symptomatic and asymptomatic individuals [57], compared to the preservation of neutralising antibodies for up to 3 months in mild/moderate COVID-19 patients [58]. Hence, there is a need to address this issue in longitudinal studies to predict whether SARS-CoV-2 will become a recurring public health challenge going forward and in the post vaccine era.

## Supporting information

S1 Raw data (ZIP)

S1 Fig Correlates of RBD:ACE-2 binding inhibition amongst cohort members demonstrating rapid humoral responses (<7 days). The relationship between anti-S1 IgG, anti-NP IgG or anti-RBD IgG development and sRBD:ACE-2 inhibition potency was investigated by Pearson’s correlation test and two-tailed p test. All *y*-axis are % RBD:ACE-2 inhibition reported by the sVNT assay, whilst *x*-axis are ratios for EuroImmun assay and Abbott assay, or OD readouts from the RBD IgG ELISA assay. *r*^2^ describes correlation coefficients. Abbreviations/symbols: ns = no significance; * = p ≤ 0.05; ** = p ≤ 0.01; *** p ≤ 0.001; **** p ≤ 0.0001.
(TIF)

S2 Fig Correlates of RBD:ACE-2 binding inhibition amongst cohort members with delayed humoral responses (between 7–14 days). Correlations between anti-S1 IgG, anti-NP IgG or anti-RBD IgG development and sRBD:ACE-2 inhibition evolution was investigated by Pearson’s correlation test and two-tailed p test. All *y*-axis are % RBD:ACE-2 blockage from the sVNT assay, whilst *x*-axis are ratios for EuroImmun assay and Abbott assay, or OD readouts from the RBD IgG ELISA assay. *r*^2^ describes correlation coefficients. Abbreviations/symbols: ns = no significance; * = p ≤ 0.05; ** = p ≤ 0.01; *** p ≤ 0.001; **** p ≤ 0.0001.
(TIF)

S3 Fig Correlates of RBD:ACE-2 inhibition amongst the remaining cohort members. The relationship between anti-S1 IgG, anti-NP IgG or anti-RBD IgG development and sRBD:ACE-2 inhibition potency was investigated by Pearson’s correlation test and two-tailed p test. All *y*-axis are % RBD:ACE-2 blockage from the sVNT assay, whilst *x*-axis are ratios for EuroImmun assay and Abbott assay, or OD readouts from the RBD IgG ELISA assay. *r*^2^ describes correlation coefficients. Abbreviations/symbols: ns = no significance; * = p ≤ 0.05; ** = p ≤ 0.01; *** p ≤ 0.001; **** p ≤ 0.0001.
(TIF)

S1 Table Documented comorbidities for the cohort members. DM—Diabetes Mellitus; CVD—Cardiovascular Disease; PVD—Peripheral Vascular Disease; Ca–Cancer; COPD—Chronic Obstructive Pulmonary Disease; AF—Atrial Fibrillation; HTN–Hypertension; MI—Myocardial Infarct; HIV—Human Immunodeficiency Virus; CLL—Chronic Lymphoid Leukaemia; IPF—Idiopathic Pulmonary Fibrosis; CKD—Chronic Kidney Disease. ^a^Y = yes, N = no. ^b^n(%)–number(percentage).
(PDF)
