# Supplementary figures and images for: Temporal development and neutralising potential of antibodies against SARS-CoV-2 in hospitalised COVID-19 patients: An observational cohort study
Source: PLoS One. 2021 Jan 26;16(1):e0245382. doi: 10.1371/journal.pone.0245382 (PMC7837461; doi:10.1371/journal.pone.0245382)

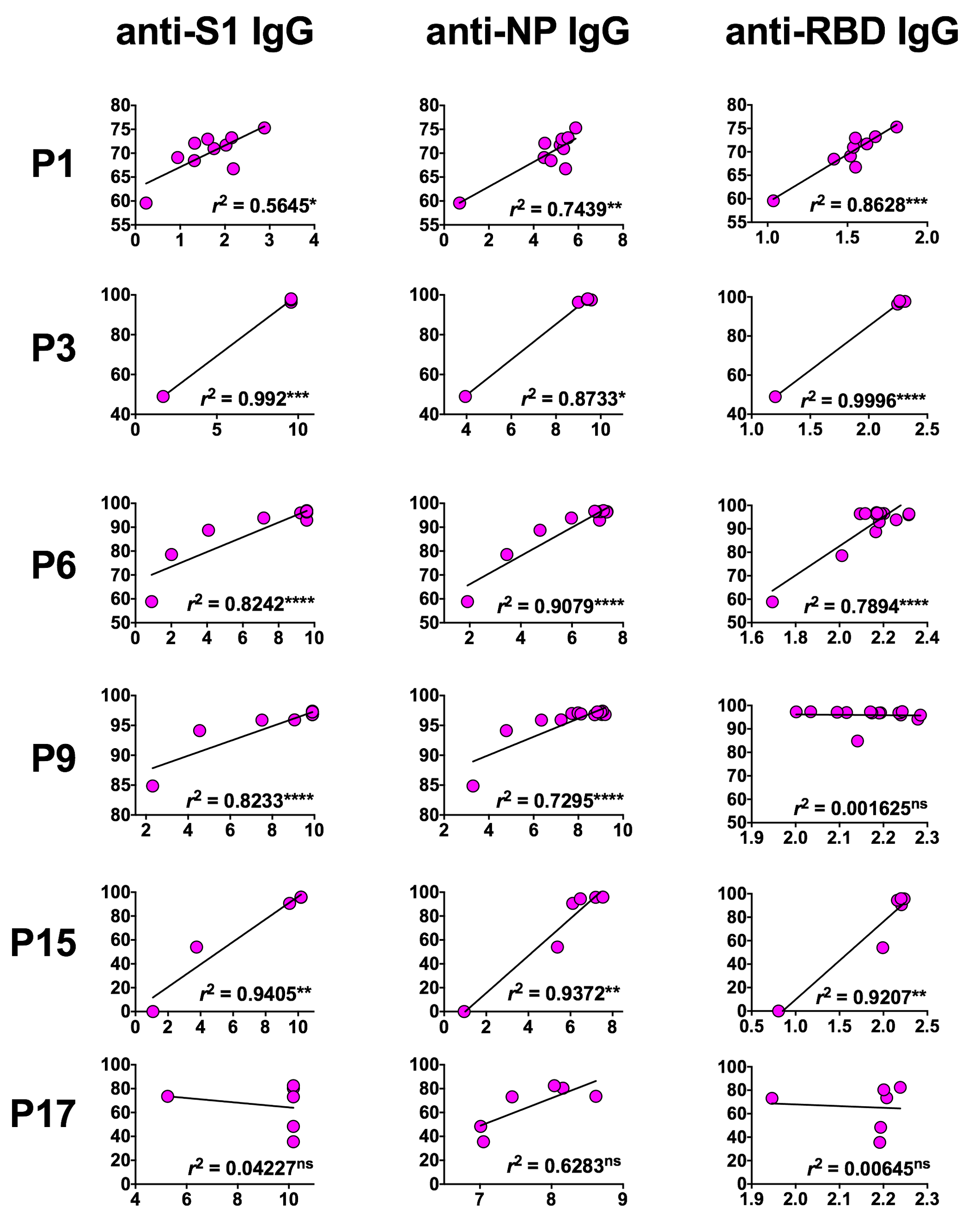

Supplement: S1 Fig — The relationship between anti-S1 IgG, anti-NP IgG or anti-RBD IgG development and sRBD:ACE-2 inhibition potency was investigated by Pearson’s correlation test and two-tailed p test. All y-axis are % RBD:ACE-2 inhibition reported by the sVNT assay, whilst x-axis are ratios for EuroImmun assay and Abbott assay, or OD readouts from the RBD IgG ELISA assay. r2 describes correlation coefficients. Abbreviations/symbols: ns = no significance; * = p ≤ 0.05; ** = p ≤ 0.01; *** p ≤ 0.001; **** p ≤ 0.0001. (TIF) [file pone.0245382.s002.tif]

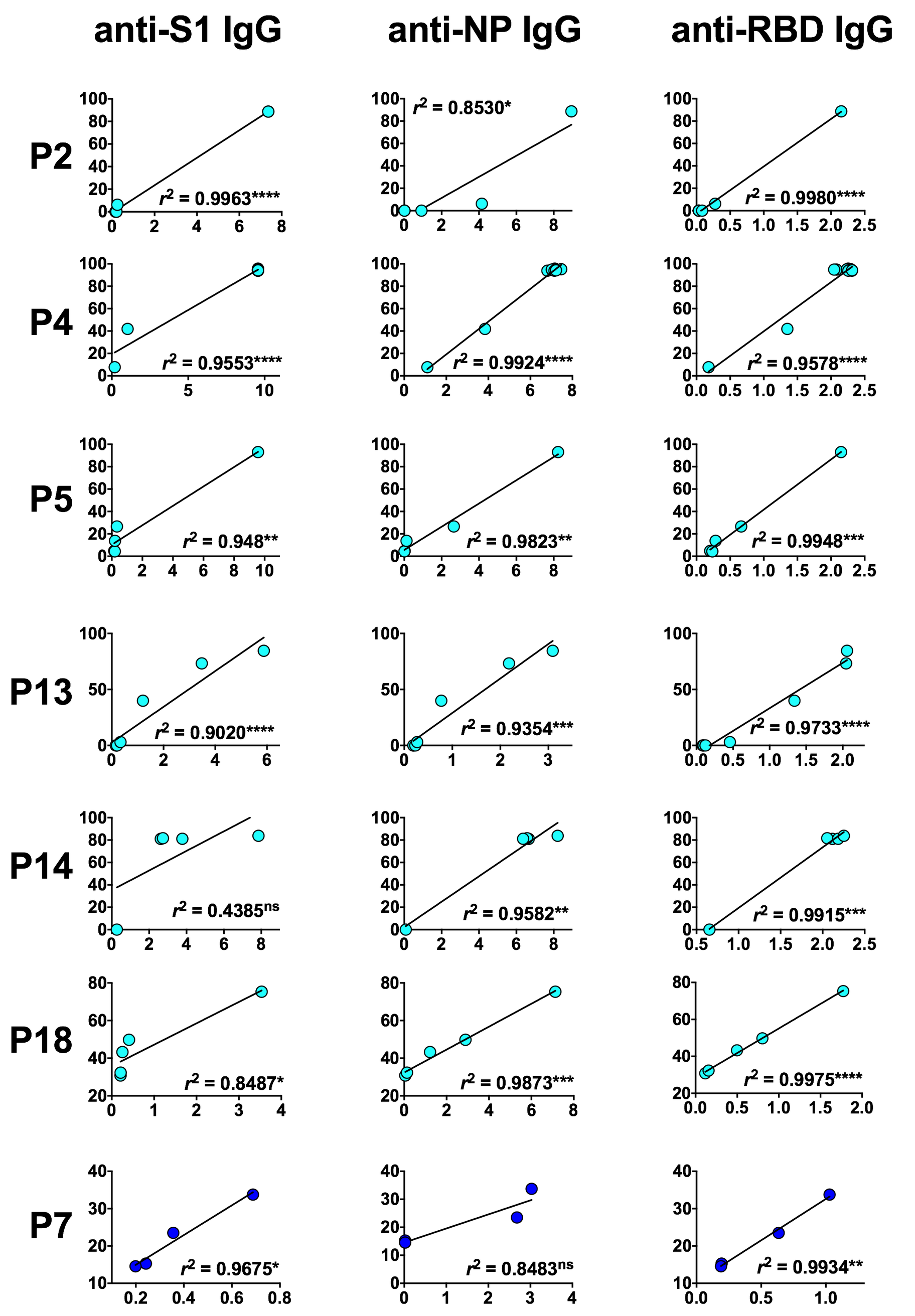

Supplement: S2 Fig — Correlations between anti-S1 IgG, anti-NP IgG or anti-RBD IgG development and sRBD:ACE-2 inhibition evolution was investigated by Pearson’s correlation test and two-tailed p test. All y-axis are % RBD:ACE-2 blockage from the sVNT assay, whilst x-axis are ratios for EuroImmun assay and Abbott assay, or OD readouts from the RBD IgG ELISA assay. r2 describes correlation coefficients. Abbreviations/symbols: ns = no significance; * = p ≤ 0.05; ** = p ≤ 0.01; *** p ≤ 0.001; **** p ≤ 0.0001. (TIF) [file pone.0245382.s003.tif]

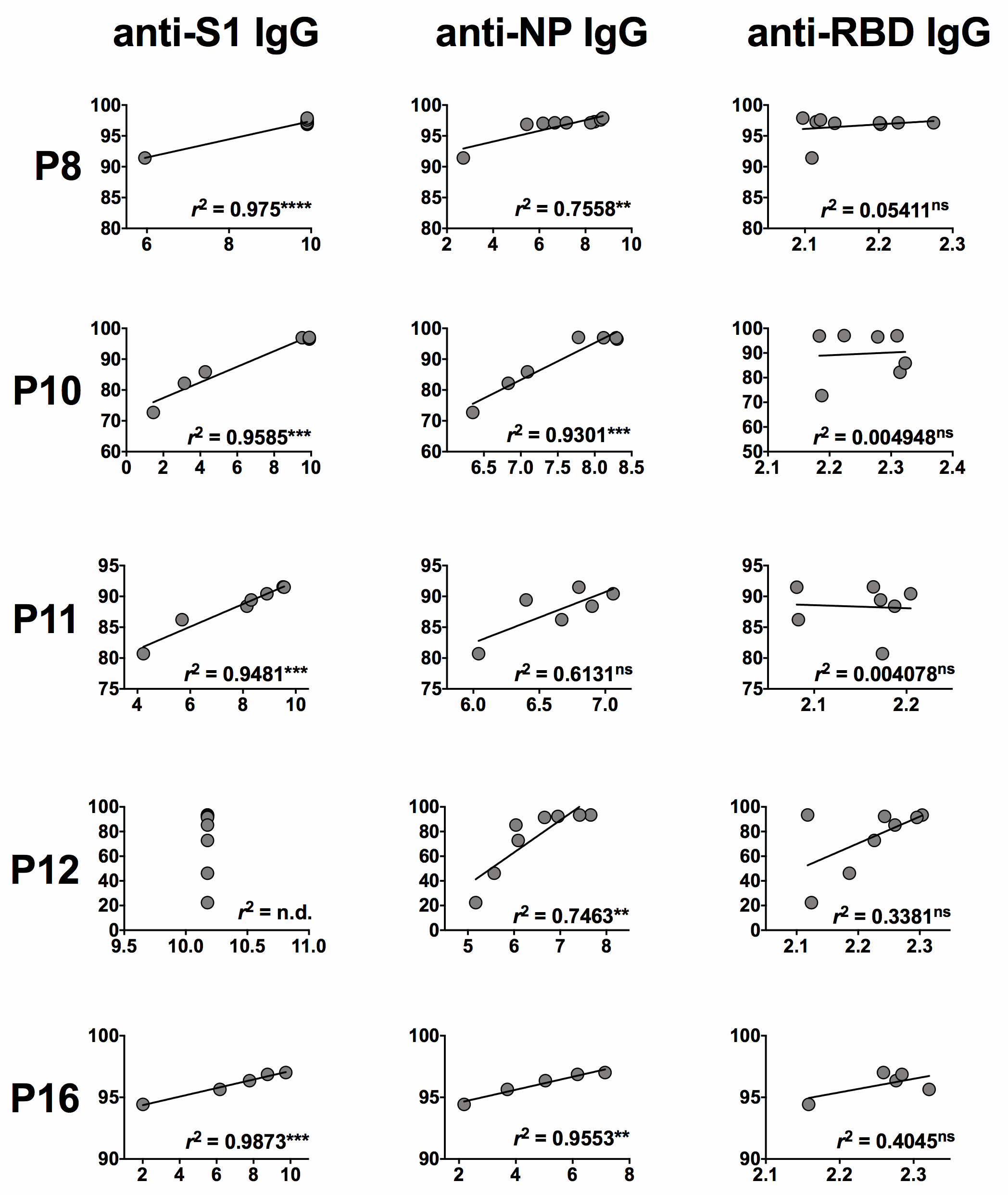

Supplement: S3 Fig — The relationship between anti-S1 IgG, anti-NP IgG or anti-RBD IgG development and sRBD:ACE-2 inhibition potency was investigated by Pearson’s correlation test and two-tailed p test. All y-axis are % RBD:ACE-2 blockage from the sVNT assay, whilst x-axis are ratios for EuroImmun assay and Abbott assay, or OD readouts from the RBD IgG ELISA assay. r2 describes correlation coefficients. Abbreviations/symbols: ns = no significance; * = p ≤ 0.05; ** = p ≤ 0.01; *** p ≤ 0.001; **** p ≤ 0.0001. (TIF) [file pone.0245382.s004.tif]
