# Supplementary material for: Temporal development and neutralising potential of antibodies against SARS-CoV-2 in hospitalised COVID-19 patients: An observational cohort study
Source: PLoS One. 2021 Jan 26;16(1):e0245382. doi: 10.1371/journal.pone.0245382 (PMC7837461; doi:10.1371/journal.pone.0245382)
Supplement: S1 Table — DM—Diabetes Mellitus; CVD—Cardiovascular Disease; PVD—Peripheral Vascular Disease; Ca–Cancer; COPD—Chronic Obstructive Pulmonary Disease; AF—Atrial Fibrillation; HTN–Hypertension; MI—Myocardial Infarct; HIV—Human Immunodeficiency Virus; CLL—Chronic Lymphoid Leukaemia; IPF—Idiopathic Pulmonary Fibrosis; CKD—Chronic Kidney Disease. aY = yes, N = no. bn(%)–number(percentage). (PDF) [file pone.0245382.s005.pdf]

	Comorbidity (Y/N)[a]	Details
Patient 1	Y	DM, CVD-PVD, Previous Ca
Patient 2	Y	COPD
Patient 3	Y	CVD
Patient 4	Y	DM
Patient 5	Y	CVD, AF, HTN
Patient 6	Y	Asthma
Patient 7	Y	CVD
Patient 8	Y	CVD, MI
Patient 9	N	-
Patient 10	Y	DM, Morbid obesity
Patient 11	Y	HIV
Patient 12	Y	AF, CLL, COPD
Patient 13	N	-
Patient 14	Y	HTN, IPF
Patient 15	Y	CKD, HTN
Patient 16	Y	Hypothyroid
Patient 17	Y	Ca, AF, HTN
Patient 18	Y	DM, CVD
Patient 19	Y	AF
	Summary **n(%)[b]**	
	3(16)/16(84)	

Details
DM, CVD-PVD, Previous
COPD
CVD
DM
CVD, AF, HTN
Asthma
CVD
CVD, MI
-
DM, Morbid obesity
HIV
AF, CLL, COPD
-
HTN, IPF
CKD, HTN
Hypothyroid
Ca, AF, HTN
DM, CVD
AF

Details	
DM, CVD-PVD, Previous Ca	
COPD	
CVD	
DM	
CVD, AF, HTN	
Asthma	
CVD	

CVD, MI	
-	
DM, Morbid obesity	
HIV	
AF, CLL, COPD	
-	
HTN, IPF	
CKD, HTN	
Hypothyroid	
Ca, AF, HTN	
DM, CVD	
AF	
